# Chinese HSCT patients' perceptions and experiences of nutrition management during transplant-unit hospitalization: a qualitative study

**DOI:** 10.3389/fnut.2026.1970435

**Published:** 2026-10-20

**Authors:** Yuqi Wang, Qiuqin Cheng, Minfang Zhu, Hangying Xu, Nafei Tu, Jiazhen Zhu, Xiaolan Zhang

**Affiliations:** 1 School of Nursing, Zhejiang Chinese Medical University, Hangzhou, Zhejiang, China; 2 The First Affiliated Hospital of Zhejiang Chinese Medical University (Zhejiang Provincial Hospital of Chinese Medicine), Hangzhou, Zhejiang, China; 3 Zhejiang Business College, Hangzhou, Zhejiang, China

**Keywords:** hematopoietic stem cell transplantation, nutrition management, nutrition-impact symptoms, qualitative study, transplant unit

## Abstract

**Background:**

Malnutrition and inadequate oral intake are common during hematopoietic stem cell transplantation (HSCT), yet little is known about how patients perceive and experience nutrition management during transplant-unit hospitalization.

**Objective:**

To explore Chinese HSCT patients' perceptions and experiences of nutrition management during transplant-unit hospitalization.

**Methods:**

A descriptive qualitative study was conducted. 16 HSCT patients were recruited using purposive and maximum variation sampling and completed semi-structured interviews after transplantation. Data were analyzed inductively using conventional content analysis, followed by clinically relevant interpretation informed by interpretive description.

**Results:**

Five themes were identified. Participants recognized nutrition as important, but often held low expectations for nutritional recovery and accepted reduced intake during hospitalization. The transplant-unit environment reduced control over eating through disrupted routines, dietary restrictions, unfamiliar food practices, and mismatched meal patterns. Nutrition-impact symptoms (NIS) not only limited intake, but also changed the meaning of food. Patients sought to regain some control through pragmatic adaptation and expressed preferences for nutritional care that was practical and symptom-adapted, family-inclusive and culturally meaningful, and aligned with realistic recovery timelines.

**Conclusions:**

Nutrition management during transplant-unit hospitalization was experienced less as a route to recovery than as a constrained effort to sustain minimal intake. These findings suggest that nutritional care in HSCT may benefit from being more symptom-integrated, family-attentive, culturally informed, and practically responsive to patients' lived realities.

## 1 Introduction

Hematopoietic stem cell transplantation (HSCT) is a procedure in which patients receive conditioning therapy (typically high-dose chemotherapy, with or without total body irradiation) to ablate the diseased marrow and suppress the immune system, followed by infusion of autologous or allogeneic hematopoietic stem cells to restore hematopoiesis and immune function (1–3). It has become one of the most effective treatments for a wide range of malignant and non-malignant hematological diseases, and its use continues to expand. In the Asia-Pacific region, 31,880 and 36,505 HSCT procedures were performed in 2022 and 2023, respectively; of these, 18,218 (57.1%) and 21,724 (59.5%) were conducted in China (4).

Malnutrition is common among patients undergoing HSCT and remains an important clinical concern. More than 55% of patients have been reported to experience substantial weight loss and a decline in body mass index (BMI) between transplantation and hospital discharge (5, 6). Malnutrition has been associated with adverse outcomes, including relapse, prolonged length of stay, delayed engraftment, increased infection risk, reduced survival, and higher healthcare costs (7–9). Therefore, it is necessary to attach importance to the nutrition of HSCT patients.

The transplant-unit phase may be particularly challenging from a nutritional perspective. Patients undergoing HSCT are admitted to a laminar airflow, HEPA-filtered, single-occupancy room under positive pressure, where they remain in relative isolation for weeks. Every object, including food, is sterilized or screened before entry, and family contact is severely restricted (10). During this period, patients commonly experience inadequate oral intake, weight loss, and multiple symptoms that interfere with eating. Treatment-related toxicities, including nausea, vomiting, taste alteration, mucositis, diarrhea, and impaired absorption, may directly reduce both the ability and the desire to eat, thereby increasing the risk of nutritional deterioration (11–13). In addition, protective isolation may negatively affect patients' psychological wellbeing and sense of control (14). These nutrition-impact symptoms (NIS) may further contribute to reduced oral energy and protein intake (15, 16).

Despite the clinical importance of nutritional support during HSCT, current nutritional guidelines are based largely on evidence generated in Western settings, whereas dedicated research in Asian transplant contexts remains limited. In addition, nutritional plans are often designed around ideal intake targets and may not always reflect the lived realities of patients, and their delivery is further complicated by variation in clinical practice, uneven guideline uptake, and the challenge of integrating nutritional care into already complex treatment routines (17–20). Although the prevalence and consequences of malnutrition in HSCT are well documented, few qualitative studies have examined how patients themselves experience and manage nutrition during the transplant-unit phase, particularly in Asian settings. In China, nutrition is often understood not only in biomedical terms but also through cultural frameworks of food therapy, nourishing (tonic) foods, dietary taboos, and family-led meal preparation, which may further shape patients' expectations and experiences of nutritional care (21). Yet little is known about what facilitates or hinders adherence to nutritional guidance in this specific context.

This qualitative study therefore aimed to explore Chinese HSCT patients' perceptions and experiences of nutrition management in the transplant unit, and to identify the barriers, facilitating factors, and support needs relevant to developing practical and culturally appropriate nutritional care strategies.

## 2 Methods

### 2.1 Study design

This study used a descriptive qualitative design to explore HSCT patients' perceptions and experiences of nutrition management during transplant-unit hospitalization (22). Although the study was grounded in a descriptive qualitative framework, its aim extended beyond summarizing participants' views to include interpretation of the meanings and clinical relevance of their experiences. Therefore, principles of interpretive description were incorporated into the analytic process to support a more in-depth and practice-relevant understanding of nutrition management in the transplant-unit context (23). The study was reported in accordance with the Consolidated criteria for reporting qualitative research (COREQ) checklist (24) (Appendix 1 in Supplementary materials).

### 2.2 Setting and participants

Participants were recruited from a tertiary teaching hospital in China between February and June 2026 through purposive sampling based on review of clinical records. To enhance heterogeneity in both demographic and clinical characteristics, maximum variation sampling was applied across age, gender, educational attainment, and marital status. The inclusion criteria included (1) having received hematopoietic stem cell transplantation, (2) no recurrence, (3) aged at least 16 years at the time of HSCT and at least 18 years old when interviewed (25, 26), (4) ability to understand and speak Chinese, and (5) provision of written informed consent. The exclusion criteria included psychiatric disease, cognitive impairment, or severe physical impairments.

Nineteen HSCT patients were approached, and three declined participation because they were not interested, leaving a final sample of 16 participants. Ongoing analysis after the first 12 interviews suggested that no substantially new information was emerging. Four additional interviews were then conducted to confirm and refine the developing thematic structure. Because these later interviews did not yield new themes, the research team determined that data saturation had been achieved (27).

### 2.3 Data collection

The first author, the sole interviewer, was a master's nursing student in China at the time of data collection. Before each interview, the researcher explained the purpose and significance of the study to the participant and obtained written informed consent. Interviews were conducted in a quiet teaching room or a single-occupancy patient room chosen by the participant, to minimize external distractions and create a comfortable environment. All interviews were audio-recorded with the participants' consent. During each interview, the interviewer took brief notes and, immediately afterward, completed detailed field notes documenting contextual observations, the interview setting, and participants' nonverbal behaviors. The average interview length was 27 min. All interviews were conducted entirely in Mandarin. A semi-structured interview guide was used and refined iteratively throughout data collection, based on ongoing review of completed interviews, to develop follow-up and probing questions.

Sample interview questions included: “How did you perceive nutrition during your hospitalization in the transplant unit?”, “What were your experiences of nutrition management during that time?”, “What nutritional needs did you have during transplant-unit hospitalization?”, and “Which factors facilitated or prevented you from adhering to nutritional guidance in that period?” Probing prompts were used to elicit more detailed descriptions (e.g., “Why was that important to you?”). The full interview guide is provided in Supplementary material (Appendix 2). Follow-up phone calls were conducted with several participants to clarify ambiguous or inaudible content in the recordings or transcripts. Raw data comprised verbatim transcripts of all face-to-face interviews, which were reviewed independently by the two researchers.

### 2.4 Data analysis

Interviews were transcribed verbatim within 24 h of completion, and analysis proceeded concurrently with data collection. Data were analyzed inductively using conventional content analysis (28). Two researchers trained in qualitative methods (Y.Q.W. and H.Y.X.) repeatedly read the transcripts to gain a holistic understanding of participants' accounts. They then performed line-by-line manual coding of all narrative material concerning perceptions and lived experiences related to nutrition management during transplant-unit hospitalization, using primarily semantic coding. No predefined coding framework was used; instead, initial codes were derived directly from the data, grouped into broader categories, and iteratively refined into subthemes and themes through constant comparison across transcripts. Representative quotations were selected to illustrate the main findings.

The research team met regularly to compare coding, revise developing categories and themes, and resolve discrepancies through discussion, with consultation from the corresponding author (X.L.Z.) when needed. All analysis was conducted manually without software support. Chinese was used for the initial coding, and the codes were subsequently translated into English for further comparison by a peer reviewer and for report writing (29, 30). The bilingual first author checked and refined the translations to ensure fidelity to the original Chinese accounts, and all co-authors reviewed the final themes, subthemes, and representative quotations.

### 2.5 Rigor and reflexivity

Several strategies were used to enhance rigor (31, 32). Credibility was supported by rapport building with participants, consensus meetings to resolve disagreements on themes and subthemes, and peer debriefing to consider alternative interpretations and methodological issues (33, 34). Dependability was supported by checking all transcripts against the audio recordings and by independent dual coding (Y.Q.W. and H.Y.X.) with discrepancies resolved through discussion. A standardized translation–back-translation procedure ensured the semantic equivalence of data translated from Chinese into English.

In addition, the first author (Y.Q.W.) kept a reflexive journal documenting her preconceptions and identity. As a graduate student researching nutrition in HSCT patients, with 1 year of clinical internship in a hematology ward, she was an informed insider yet not involved in participants' care; her nationality (Chinese), health status (healthy), age (young adult), and occupation (nursing student) may also have shaped her interpretation. All researchers received qualitative-methods training and met regularly to reflect on the research process and analysis.

### 2.6 Ethical consideration

The Medical Ethical Committee of The First Affiliated Hospital of Zhejiang Chinese Medical University approved this study (no. 2025-KLS-861-02). Participants received detailed information about the study aims, procedures, confidentiality measures, and their right to withdraw at any time without consequences. Written informed consent was obtained from participants before each interview. Confidentiality was ensured, and all results are presented anonymously.

## 3 Results

Sixteen participants were interviewed. Their mean age was 48.19 years (SD = 13.17; range: 20–67 years); six were female and three unmarried. Educational attainment ranged from primary to tertiary level. At the time of interview, participants were 3–21 months post-transplantation. 14 participants had received allogeneic HSCT and two autologous HSCT; among the allogeneic recipients, 13 had related donors and one an unrelated donor from a bone marrow registry. Five participants had received reduced-intensity conditioning and 11 myeloablative conditioning. One participant had hypertension. Additional demographic and clinical characteristics are presented in Table 1. The analysis generated five themes and 17 subthemes that captured key aspects of nutrition management during transplant-unit hospitalization after HSCT (Table 2).

**Table 1:** Sociodemographic characteristics of the sample (16 patients).

ID	Age (Years)	Gender	Marital status	Education level	HSCT type	Duration after HSCT (month)	Donor type	Conditioning regimen	Diagnosis type
P1	58	male	Married	Secondary education	Allo-HSCT	10	HRD	MAC	Acute myeloid leukemia
P2	28	male	Unmarried	Tertiary education	Allo-HSCT	16	MUD	MAC	Aplastic anemia
P3	36	male	Unmarried	Secondary education	Allo-HSCT	21	HRD	RIC	Aplastic anemia
P4	64	male	Married	Secondary education	Allo-HSCT	5	HRD	MAC	Myelodysplastic syndromes
P5	47	female	Married	Primary education	Allo-HSCT	5	HRD	RIC	Aplastic anemia
P6	45	female	Married	Secondary education	Allo-HSCT	7	HRD	MAC	Aplastic anemia
P7	64	female	Married	Secondary education	Allo-HSCT	8	HRD	MAC	Acute lymphoblastic leukemia
P8	60	male	Married	Secondary education	Allo-HSCT	6	HRD	MAC	Acute myeloid leukemia
P9	40	female	Married	Secondary education	Allo-HSCT	11	HRD	MAC	Acute myeloid leukemia
P10	53	male	Married	Secondary education	Auto-HSCT	11	Autologous	MAC	Multiple myeloma
P11	20	female	Unmarried	Secondary education	Allo-HSCT	6	HRD	RIC	Aplastic anemia
P12	43	male	Married	Secondary education	Allo-HSCT	6	HRD	RIC	Aplastic anemia
P13	51	female	Married	Secondary education	Allo-HSCT	3	HRD	MAC	Myelodysplastic syndromes
P14	44	male	Married	Primary education	Allo-HSCT	15	HRD	RIC	Aplastic anemia
P15	67	male	Married	Secondary education	Allo-HSCT	12	HRD	MAC	Myelodysplastic syndromes
P16	51	male	Married	Secondary education	Auto-HSCT	14	Autologous	MAC	Multiple myeloma

Auto-HSCT, autologous HSCT; Allo-HSCT, allogeneic HSCT; MAC, myeloablative conditioning; RIC, reduced-intensity conditioning; HRD, haploidentical related donor; MUD, matched unrelated donor.

**Table 2:** Overview of themes and sub-themes.

Theme	sub-themes	Frequency	Sample statements of participants
Patients recognized the importance of nutrition but held low expectations and accepted reduced intake during transplant-unit hospitalization	Nutrition was seen as important for treatment tolerance and recovery	13/16	“I need to eat as much as possible to recover faster… to prevent my physical strength from dropping. As long as I do not vomit and can keep things down, I try to drink rice-water congee.” (p1) “When I could eat properly, I definitely felt more energetic.” (p5) “I felt I had to get food into my body so my cells could regenerate faster and my body could hold on. Otherwise, poor nutrition would slow recovery.” (p12) “I wanted to get better. Even with mouth ulcers and throat pain I forced myself to swallow food.” (p13) “Better nutrition helps the body recover gradually.” (p8)
	Patients held low expectations for nutritional improvement during hospitalization	12/16	“Nothing I eat in the transplant unit makes much difference; I just have no appetite and my abdomen is bloated.” (p15) “I just had to get through this phase; nutritional recovery would happen slowly after discharge.” (p8) “There was nothing I could do about poor oral intake in the unit; I just had to endure it.” (p4) “Little nutritional improvement could be achieved in-hospital; I would adjust my diet slowly once my gut recovered post-discharge.” (p13)
	Reduced intake was often accepted as a reality of the transplant-unit phase	11/16	“I just could not eat, and weight loss followed. This was normal for the transplant unit. If I could not eat, I simply did not eat.” (p11) “Nothing I ate made much difference in the transplant unit. I simply had no appetite and suffered abdominal bloating. I just had to get through this period.” (p15) “Once chemotherapy started, I completely lost my appetite. There was nothing I could do; this was just how life was inside the transplant unit.” (p4) “Even though I wanted to eat, my physical condition prevented it, so I had to accept eating very little.” (p8) “Sometimes I could not manage even one mouthful. Many people in the transplant unit went through the same thing.” (p15)
The transplant-unit context reduced patients' sense of control over eating	Disrupted routines and persistent exhaustion weakened eating capacity	9/16	“I barely slept for over a month. Intravenous infusions ran around-the-clock and constant interruptions prevented rest. Whenever I wanted to sleep, something would come up.” (p6) “I felt lethargic and mentally foggy. I lacked energy to read educational handouts or focus on eating.” (p8) “Multiple intravenous lines limited mobility; I felt constantly worn-out.” (p4) “I had very little physical strength and no energy for proper meals.” (p15) “Physical consumption was high in the unit.” (p7)
	Dietary restrictions reduced autonomy over food choice	16/16	“Rich tonic foods such as eel, soft-shelled turtle and ginseng were forbidden; recovery had to proceed step-by-step.” (p1) “There were foods I wanted to eat, yet if doctors or nurses advised against them, I would avoid them.” (p2) “Many desirable foods were off-limits; the dishes I craved could not be prepared for me.” (p6) “Marine fish was prohibited; only freshwater fish was permitted. Sea cucumber and loach were forbidden; high-tonic foods were temporarily restricted.” (p13) “Spicy food was banned; many of my preferred strong-flavored dishes were not allowed.” (p14)
	Meal patterns further reduced food acceptability	8/12	“Meal times were fixed. Sometimes I got hungry long before the next meal... I had to wait until the official mealtime.” (p2) “Family-cooked meals were disinfected in the microwave before delivery. Repeated reheating ruined the texture.” (p1) “My family lived far away. Food turned soggy during transport and became even less appetizing.” (p7) “Meals were low-oil and low-salt, resulting in very bland flavors.” (p6)
Nutrition-impact symptoms progressively undermined both the controllability and the meaning of eating	Nausea, vomiting, and taste changes reduced food tolerability	11/16	“The smell of meat alone made me nauseous; steamed egg custard would trigger vomiting.” (p8) “My sense of taste was blunted; I could barely distinguish sweet or salty flavors.” (p4) “Chemotherapy altered taste perception and dulled flavor sensation.” (p12) “The sight of food could make me feel repelled; certain food odors suppressed my appetite.” (p9) “Chemotherapeutic agents induced nausea and diminished enjoyment of food.” (p12) “Food consistently tasted bitter throughout parts of my hospital stay.” (p7)
	Mucositis and swallowing pain created a gap between hunger and the ability to eat	12/16	“I suffered from oral mucositis. My mouth hurt when I opened it, even though I craved soft fish.” (p1) “During episodes of mouth ulceration my throat was sore. Although I felt hungry, I had to force myself to swallow food.” (p13) “Mouth ulcers hurt even without eating; they forced me to eat only small amounts.” (p15) “My gums were swollen and my gum tissue was breaking down.” (p6) “I did get mouth ulcers back when I was in the transplant unit… It hurt even when I wasn't eating anything…” (p5)
	Diarrhea, bloating, and poor absorption undermined confidence in maintaining intake	14/16	“Eating would trigger bloating and vomiting if I forced food down.” (p7) “Even small food portions caused stomach pain, after which I could no longer eat.” (p9) “Food passed straight through with diarrhea; I doubted whether nutrients were being absorbed.” (p13) “Gurgling intestines and recurrent diarrhea made me afraid to eat for fear of worsening gut symptoms.” (p16) “Oral nutritional supplement powder triggered diarrhea so I could not use it.” (p15)
Patients sought to regain some control over eating	Patients prioritized tolerable foods over ideal nutritional targets	10/16	“Potato noodles and cold starch sheets were soft, suited for mouth ulcers and provided some nutrition. Steamed egg custard was eaten every day.” (p1) “I could only eat rice congee and millet congee; other foods were intolerable and there was nothing I could do.” (p8) “During diarrhoeal episodes I ate egg custard first and avoided meat; I re-introduced meat only after gut improvement.” (p13) “When my stomach felt sensitive I began with rice-water congee to condition my intestines before adding other foods.” (p16) “I focused on congee and noodles that I could tolerate.” (p6)
	They adjusted eating pace, amount, and expectations to sustain minimal intake	9/16	“At my worst I could not eat solid food. I refused to send my wife's meals back untouched and drank the soup and congee broth to sustain myself.” (p1) “I ate slowly, distracted myself, and tried to consume a little more bit by bit.” (p12) “I reduced portions when I could not manage more; I would stop eating before vomiting occurred.” (p11) “Even with ulcer-related pain I forced small swallows just to maintain minimal food intake.” (p13) “I did not aim for large meals; my goal was just to prevent rapid physical decline.” (p14)
	They relied on family and professional support to negotiate eating in practice	7/16	“I told my wife I wanted soft-textured winter melon and steamed egg custard.” (p1) “I got fed up with the food, so I asked my husband to make noodles to change things up.” (p5) “If there was something I felt like eating, I would first ask the nurses or doctors whether I could have it. If they said no, I wouldn't eat it.” (p2)
Patient aspirations and preferences regarding nutritional care	Retaining some autonomy over food-related decisions	8/16	“I told my wife never to cook radish for me in the unit… I preferred softer foods such as winter melon.” (p1) “When I grew tired of certain dishes I asked my family to switch to noodles for variety.” (p5) “I discussed meal-planning with my family, including when to use nutritional powder and which groceries to buy.” (p12)
	Receiving practical, symptom-adapted guidance	13/16	“The handbook said I could eat certain things, but in reality I really couldn't. When I asked the doctor, they told me I shouldn't eat them.” (p6) “What was written in the handbook didn't always match real-life situations. It depended on what symptoms I was having at the time.” (p4) “During diarrhea doctors told me to avoid meat temporarily and eat egg custard, reintroducing meat only after gut recovery.” (p13) “My attending physician suggested tomato-based soups to stimulate appetite when I could not eat.” (p16)
	Involving family members in nutritional support	14/16	“My family kept encouraging me to keep eating, even when I felt really unwell. I forced myself to finish my meals for them.” (p5) “My husband learned how to cook just for me. He would blanch ingredients first and then cook them again to make everything clean and safe.” (p13)
			“It was my mum and my sister who prepared my food. I am very grateful to them.” (p2) “My wife took care of all the cooking for me while I was in the unit.” (p1)
	Aligning advice with culturally familiar food meanings and recovery beliefs	8/16	“Older generations believe glutinous rice soothes the stomach, so we cooked congee combining ordinary rice and glutinous rice.” (p1) “The folk saying ‘eat radish in winter and ginger in summer' applies to ordinary health, not transplant-unit patients.” (p1) “Traditional folk belief considers sweet potato, pumpkin and taro mild and restorative.” (p12) “Strong tonic foods including eel, soft-shelled turtle and ginseng were prohibited early post-transplant; nutritional tonification needed gradual progression.” (p15) “Local folk belief regards duck eggs as milder than chicken eggs, so duck eggs were chosen for protein supplementation.” (p16)
	Establishing realistic timelines for nutritional recovery	11/16	“Before entering the unit I learned that losing around 20 jin was normal for transplant patients.” (p1) “My doctor told me body weight would likely recover only after approximately one-year post-transplant.” (p9) “I needed to endure dietary restrictions in-hospital. After discharge and gut healing I could gradually resume richer foods without rushing.” (p13) “Appetite recovery after transplantation took time; meaningful improvement came roughly 2 months post-discharge.” (p15) “Rapid heavy tonification was discouraged; recovery had to proceed step-by-step to avoid overwhelming the body.” (p1)

### 3.1 Patients recognized the importance of nutrition but held low expectations and accepted reduced intake during transplant-unit hospitalization

Thirteen of the 16 participants consistently described nutrition as vital to maintaining strength, tolerating treatment, and promoting recovery. Eating was often framed as necessary for “supporting the body” and preventing further physical decline. For example, one participant remarked, “Eat more nourishing food, and you'll recover faster. Poor nutrition will leave your body weak; if you eat and sleep well, you'll heal properly” (P5), while another stated that “if your nutrition is insufficient, you'll lack strength” (P8). A third participant similarly explained, “I always feel I have to eat something so my cells can regenerate faster and my body can hold up. Otherwise, without adequate nutrition… the less you eat, the slower your recovery will be, and your physical condition will only get worse” (P12).

However, recognizing the importance of nutrition did not necessarily mean that participants expected it could be meaningfully improved during hospitalization. Rather than viewing this phase as a time to actively restore nutritional status, many (*n* = 12) saw it as a period to get through with whatever intake remained possible. One participant commented, “Nutrition? There's hardly any nutrition in the transplant unit… Even if I wanted better nutrition, it would be pointless” (P10), while another said, “I don't expect much in terms of nutrition while staying in the transplant unit… we're all in the same boat” (P4).

When eating became difficult, participants often lowered their expectations and accepted reduced intake as part of the transplant-unit phase. Rather than expressing intense emotional conflict, they (*n* = 11) more commonly conveyed a pragmatic acceptance of eating less as an unavoidable consequence of treatment, symptoms, and the inpatient environment. As one participant explained, “after a few bites I push the plate away and stop eating… All I cared about was getting better” (P10), while another noted, “if I can't stomach it, I just leave it… anytime nausea hit, I wouldn't eat another bite” (P11).

### 3.2 The transplant-unit context reduced patients' sense of control over eating

A recurrent feature of nine participants' accounts was the disruption of daily rhythm. Continuous infusions, frequent toileting, repeated interruptions, and the procedural demands of hospitalization left many participants sleep-deprived and persistently exhausted. Poor sleep and overall depletion were described as indirectly affecting appetite, energy, and tolerance for eating. One participant explained, “I couldn't get any sleep for 3 or 4 days straight while I was in the transplant unit… I barely slept at all during that month… Each day felt like a full work shift, with constant interruptions and demands” (P6). One participant stated that staying in the transplant unit had “drained all my energy” and left them “too weak to eat anything” (P8).

Food choice was also increasingly constrained after entering the transplant unit. Participants (*n* = 16) described losing control over what they could eat, especially when desired foods were either prohibited or allowed only in very small amounts. One participant explained that, because of diarrhea, the doctor advised avoiding meat and limiting intake to steamed egg custard until gastrointestinal symptoms improved (P13). Another participant similarly noted that, due to intestinal sensitivity, any new food had to be introduced “tiny bit by bit, just like you would for a baby” (P9). Food during transplant-unit hospitalization often felt disconnected from participants' usual taste preferences and eating habits. One participant noted that people from Jiangxi were “naturally fond of chili peppers” (P14), while another explained that without chili, foods such as fish or meat became “more rank and flavorless” (P15).

Eight participants also described a mismatch between the meal patterns they were used to at home and the unfamiliar institutional meal structure. One participant explained, “My appetite was very small. I could only manage a few bites at each main meal, but then I would get hungry again well before the next mealtime. I just wanted a few snacks to tide me over, but there were only three fixed meals a day in the unit—no food at all between meals.” (P9). Another participant added that solitary eating further diminished the experience: “At home, we always ate together as a family. Here, I ate alone. It felt cold and empty. Every meal just felt like a task to get through” (P13).

### 3.3 NIS progressively undermined both the controllability and the meaning of eating

Nausea, vomiting, and taste changes were commonly described as making food difficult to tolerate. Participants (*n* = 11) reported that food smells could provoke nausea and that previously acceptable foods became intolerable. Taste changes also altered the sensory experience of eating, making food seem bland, unpleasant, or indistinguishable. One participant explained, “I just can't taste anything properly. Whether it's sweet or salty… everything tastes the same. It's extremely bland, like no seasoning was added at all” (P1). Another participant described a strong smell-triggered reaction, stating that “the smell of meat alone makes me throw up… the scent of any food triggers nausea” (P8), while a third simply commented that the meals “just smell unpleasant” (P15).

Oral and throat symptoms, particularly mucositis and swallowing pain, directly hindered eating. Many participants (*n* = 12) described being unable even to drink water despite still feeling physically hungry. One participant recalled that after developing mouth ulcers, “for several days, I could not eat anything at all, not even plain boiled water” (P10). Another participant described a painful disconnection between hunger and the ability to eat, explaining, “My mouth tasted bitter… yet my stomach was empty and hungry. If I could just swallow the food and get it down, that would help—at least some nutrition would get into my stomach” (P7).

Fourteen participants reported that gastrointestinal dysfunction, including diarrhea, bloating, impaired digestion, and poor absorption, further reduced their capacity to sustain oral intake. Participants often evaluated food not only by whether it could be swallowed, but also by whether it would worsen abdominal discomfort or fail to be absorbed. One participant reported that intestinal graft-vs.-host disease led to a 13-day period of fasting, describing it as “the worst” period of the entire hospitalization (P4). Another participant explained that persistent diarrhea meant that “almost none of the nutrients got absorbed” (P13). Severe bloating also made intake increasingly difficult; as one participant described, “Food wouldn't digest after I ate it… even plain warm water made me feel bloated” (P7).

### 3.4 Patients sought to regain some control over eating

Despite these constraints, participants were not passive recipients of nutritional care. Instead, many (*n* = 10) described actively trying to regain some control over eating by choosing foods they could tolerate and adjusting intake to what felt manageable. Greasy, strongly smelling, or irritating foods were often avoided, while softer or blander foods were preferred. As one participant explained, “Some foods would make me nauseous, so I only picked what I could tolerate, even if it was not the most nutritious” (P11).

Participants (*n* = 9) also described using behavioral strategies to maintain intake. Rather than aiming to eat normally or feel full, they lowered their expectations and focused on consuming small amounts to preserve strength. One participant recalled, “I forced myself to drink as much thin rice congee as I could as long as I didn't throw it up” (P1). One participant stated, “I would watch TV or scroll on my phone while eating, take tiny bites and go slow, so I would not focus on the nausea.” (P12).

Some participants (*n* = 7) also sought minor adjustments through communication with family members or clinical staff, such as changing food preparation or requesting substitutions. One participant described how even a simple dietary adjustment could help restore intake: “The attending doctor told me to prepare some sour foods to boost my appetite, like tomatoes. Sour things help stimulate hunger. After eating those for a while, I gradually managed to eat solid food again—before that, I couldn't swallow anything at all” (P16).

### 3.5 Patient aspirations and preferences regarding nutritional care

Participants (*n* = 8) wanted to retain at least some autonomy over food-related decisions. Although most participants accepted dietary restrictions, they hoped for flexibility in choosing foods they could tolerate. One participant noted, “I know they have to follow sterile rules here. It would just be nice if I could pick something I can actually stomach.” (P11). Another added, “I understand safety comes first, but I'd like a say in what I eat.” (P9).

Thirteen of 16 participants reported wanting nutritional guidance that was practical and responsive to current symptoms. General advice such as “eat more” felt too vague when patients were dealing with nausea, mucositis, bloating, diarrhea, or poor appetite; they wanted concrete suggestions about suitable foods and how eating could be adjusted as symptoms changed. One participant commented, “Just telling me to eat if I can doesn't help when I'm constantly nauseous. Tell me what's gentle on my stomach right now.” (P4). Another explained, “It's not enough to say eat well. I need advice that changes as my symptoms get better or worse.” (P14).

Family involvement was another central expectation. Participants (*n* = 14) saw family members as essential because they understood long-standing eating habits, taste preferences, and tolerability, and because nutritional decisions were experienced as a collective responsibility. One participant described, “My family prepares food for me while I'm in the unit... We are all in this together.” (P10).

Eight participants reported that dietary advice felt more acceptable when it aligned with culturally rooted beliefs regarding food and recovery. One participant explained, “My father was a rural barefoot doctor, so our family believes in Chinese medicine food therapy. Rice congee nourishes the stomach, whereas high-dose chemotherapy harms the stomach and spleen, so red dates and Chinese yam are gentle foods that aid recovery.” (P1). Another noted, “We have always believed in Chinese medicine.” (P14).

Participants (*n* = 11) preferred realistic rather than overly optimistic timelines for nutritional recovery, finding rapid or ideal framing discouraging. One participant stated, “Don't paint a rosy picture for me. I want to know how slowly things might improve.” (P9). Another added, “I need to understand that getting back to normal eating may take a long time.” (P13).

To further synthesize the relationships among these findings, a conceptual model was developed (Figure 1). At the center of the model is patients' diminished control over nutrition management during transplant-unit hospitalization. Environmental restriction and symptom burden represent the main contextual conditions that undermined this control, while the “patients' perceptions” component captures how participants understood and articulated this experience through recognizing the importance of nutrition, holding low expectations for recovery, and accepting reduced intake as a practical reality. Against this background, patients sought to regain some limited control through tolerable foods, eating adjustment, professional guidance, and family support. The arrows represent interpretive relationships among these elements rather than fixed causal pathways.

**Figure 1:**
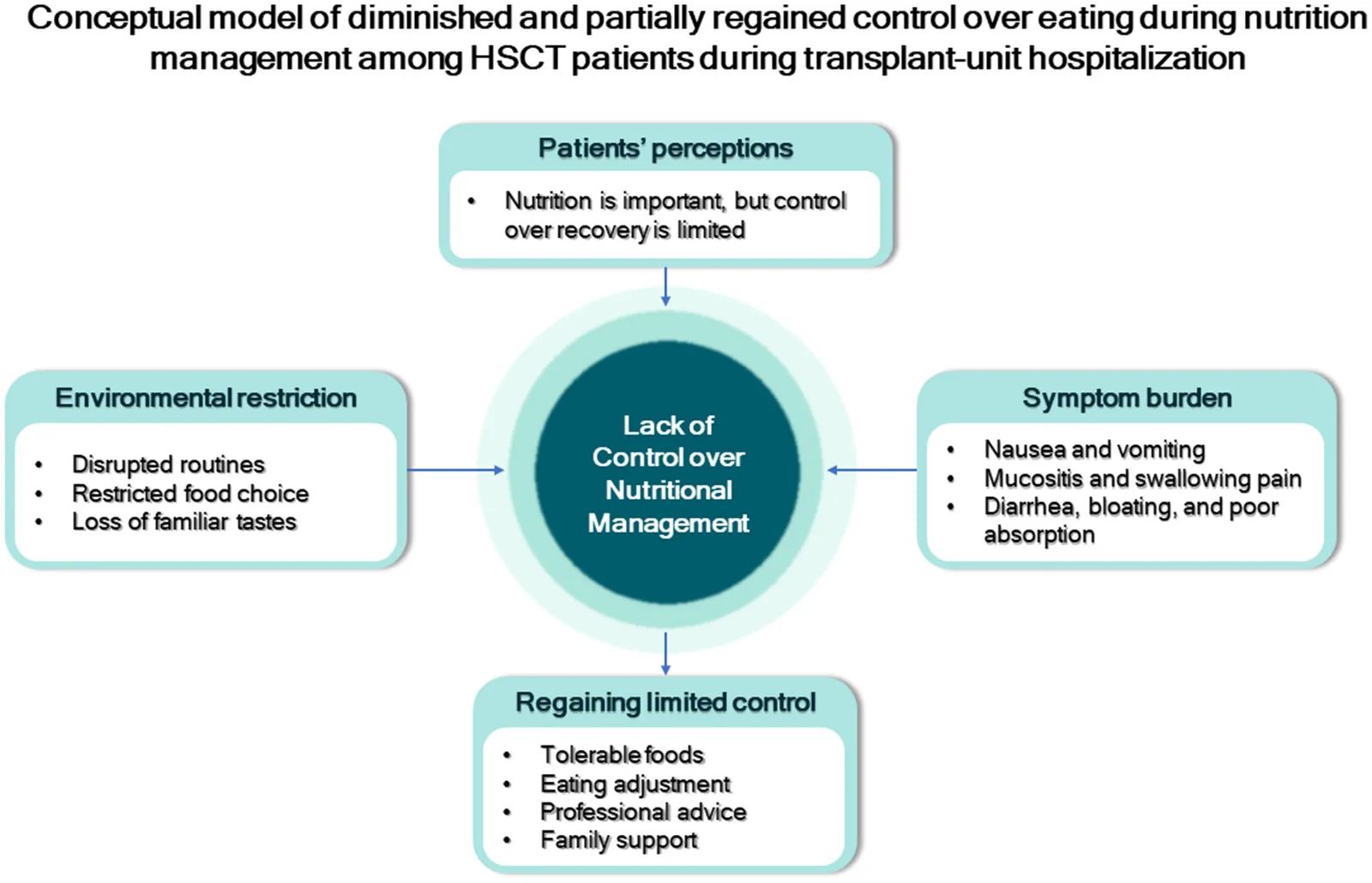
Conceptual model of diminished and partially regained control over eating during nutrition management among HSCT patients during transplant-unit hospitalization.

## 4 Discussions

This study offers a patient-centered account of nutrition management during transplant-unit hospitalization and shows that, for Chinese HSCT patients, nutrition was not experienced as a straightforward means of recovery. Rather, it became a constrained practice of sustaining the minimum intake possible under conditions of symptom burden, institutional regulation, and diminished bodily control. Existing HSCT nutrition literature has largely framed malnutrition and inadequate intake as quantifiable clinical problems requiring assessment and intervention, but has said much less about how nutritional care is actually lived by patients in the transplant unit. Our findings suggest that the central challenge was not simply that patients ate too little, but that eating had lost its meaning and capacity as a restorative act.

One of the central findings of this study was the gap between the recognized importance of nutrition and the perceived feasibility of nutritional recovery during transplant-unit hospitalization. Consistent with the study by Rodgers et al. (35), participants in our study did not dismiss nutrition as irrelevant; on the contrary, many clearly linked eating with strength, treatment tolerance, and recovery. Yet this recognition did not translate into an expectation that nutrition could be meaningfully improved during hospitalization. Instead, many appeared to defer the possibility of “real” nutritional recovery to a later phase of recovery and to understand the inpatient phase primarily as a time to endure treatment with whatever intake remained possible. This tension points to a mismatch between protocol-driven nutritional care and patients' lived realities in the transplant unit. Whereas nutritional recommendations were typically framed around ideal intake goals, patients' actual eating decisions were shaped by tolerability, acceptability, and immediate manageability under conditions of symptom burden and institutional restriction (36). In this sense, the problem was not insufficient awareness of nutritional importance, but limited capacity to translate nutritional ideals into actionable practice. Seen in this light, the difficulty of nutritional support in HSCT may be understood not simply as one of patient adherence, but as an implementation challenge.

The transplant-unit environment itself played a major role in eroding control over eating. Repeated interruptions, continuous infusions, frequent toileting, and poor sleep contributed to a state of persistent exhaustion that further reduced appetite and tolerance for intake. Sleep disturbance has been linked to dysregulation of appetite-related hormones such as leptin and ghrelin (37), and may interact with fatigue and nutritional deterioration in a mutually reinforcing cycle (38–40). Dietary restrictions further narrowed patients' room for maneuver, especially when desired foods were prohibited or allowed only in minimal amounts, consistent with previous findings of reduced food-related autonomy (41–43). Moreover, some participants indicated that a grazing pattern of small, repeated intake would have been more manageable, whereas food provision in the transplant unit remained organized around fixed mealtimes. Nutritional difficulty in the transplant unit reflects not simply insufficient intake, but a deeper mismatch between institutional feeding routines and patients' altered bodily capacity to eat at all.

NIS did not merely reduce the amount that patients could eat (44, 45); they also changed what food meant to them. Under conditions of nausea, oral pain, bloating, poor absorption, and exhaustion, food was no longer consistently experienced as nourishing, restorative, or comforting. Instead, it could become painful, aversive, uncertain, or even futile (46). This shift in meaning helps explain why many participants no longer viewed transplant-unit hospitalization as a realistic time for nutritional recovery. In other words, symptom burden altered not only eating behavior but also the symbolic and experiential value of food. From this perspective, what might appear clinically as reduced intake may be better understood as a symptom-driven redefinition of eating itself.

### 4.1 The cultural and contextual dimensions of nutritional care: Asian perspectives

The present findings suggest that nutrition management during HSCT was not experienced as a purely biomedical issue, but as a culturally embedded practice. In the Chinese context, food is rarely understood as nutrient provision alone (46). Rather, it is often linked to recuperation, bodily adjustment, and the gradual restoration of strength. This broader understanding helps explain why participants did not judge foods solely by their nutritional content, but also by whether they seemed appropriate, “clean,” safe, or helpful for recovery. Such judgments are consistent with the longstanding idea that food and medicine share a common origin, and with related dietary logics in which foods are understood to differ according to flavor, perceived warming or cooling properties, and their suitability for the body's current condition (21, 47, 48). After intensive treatment such as HSCT, symptoms including poor appetite, nausea, vomiting, and fatigue may therefore be interpreted not only as biomedical side effects, but also through a recuperative logic in which the body is seen as weakened and in need of careful adjustment. Thus, although participants' cautious and gradual approach to eating was clearly constrained by symptom burden and institutional rules, the meanings they attached to food suggest that nutrition management was also being understood through culturally familiar ideas of convalescence and bodily care.

Family involvement was another important contextual feature of nutrition management. Family members were not peripheral supporters, but active participants in preparing food, monitoring restrictions, judging what was appropriate to bring, and sustaining patients' willingness to continue eating (49–51). In this sense, nutritional care was not experienced as an individual act of self-management, but as a relational process involving patients, family members, and healthcare professionals (52). In the Chinese context, such participation likely reflects not only practical caregiving, but also a broader ethic of collective responsibility, in which illness management is commonly shared by the family rather than borne by the individual alone.

Eating also carried interpersonal and emotional meaning (53, 54). For some participants, what was disrupted in the transplant unit was not only appetite or food choice, but the social form of eating itself. Shared meals, familiar tastes, and family presence are important elements of everyday eating in many Chinese households. By contrast, solitary ward meals and rigid food routines could make eating feel cold, impersonal, and task-like. The contrast between culturally familiar shared-meal practices and the individualized, regulated meal structure of the transplant unit may therefore reduce not only food acceptability, but also the social and emotional value of eating (55). Together, these findings suggest that nutritional care in Asian transplant settings may need to move beyond generic dietary instructions toward culturally informed, family-centered communication. Clinicians may need to explore patients' and family caregivers' understandings of suitable foods, recovery trajectories, and dietary restrictions, rather than relying solely on one-size-fits-all dietary guidance. At the same time, food-related perceptions and decisions should not be assumed to be culturally uniform even within China, where regional cuisines, local taste preferences, and stage-specific beliefs about recovery may all shape what patients regard as appropriate or supportive foods (56). More broadly, these findings highlight the need for comparative research across Asian populations, where food beliefs, family caregiving roles, and recovery-related eating practices are unlikely to be homogeneous.

### 4.2 Limitations

Several limitations should be noted. First, this was a single-center study with a relatively small sample. As such, these findings are preliminary and can only provide targeted insights into nutritional management among Chinese patients receiving HSCT. Second, participants were interviewed after transplantation and were asked to recall their experiences during transplant-unit hospitalization, so recall bias is possible. Third, the interviews were relatively brief, which may have limited the depth of some accounts. Fourth, because food, family responsibility, and treatment cooperation are socially valued topics, social desirability bias cannot be excluded. Fifth, the study did not triangulate patients' accounts with those of family caregivers, nurses, physicians, or dietitians, and therefore reflects only one stakeholder perspective. Sixth, patients with the most severe symptom burden, as well as those receiving enteral or parenteral nutrition, may have been underrepresented, potentially limiting the range of nutritional experiences captured. Finally, although the findings illuminate important cultural and contextual dimensions of nutritional care in one Chinese transplant setting, their transferability to other institutions within China or to other Asian cultural contexts remains to be examined through comparative research.

## 5 Conclusion

In conclusion, this study shows that nutrition management during transplant-unit hospitalization was experienced not as a straightforward route to recovery, but as a constrained effort to sustain minimal intake under difficult conditions. Its two main contributions are the demonstration that patients experienced limited control over nutritional recovery during hospitalization, and the finding that nutrition-impact symptoms changed not only how much patients could eat, but also what food meant to them. These insights support the development of nutritional care that is more symptom-integrated, culturally attuned, and practically responsive to patients' lived realities.

## Data Availability

The original contributions presented in the study are included in the article/Supplementary material, further inquiries can be directed to the corresponding author.
