# Supplementary material for: Chinese HSCT patients' perceptions and experiences of nutrition management during transplant-unit hospitalization: a qualitative study
Source: Front Nutr. 2026 Oct 20;13:1970435. doi: 10.3389/fnut.2026.1970435 (PMC13727599; doi:10.3389/fnut.2026.1970435)
Supplement: Supplementary file 1 [file Table_1.docx]

**Appendix 1: Consolidated criteria for reporting qualitative studies (COREQ): 32-item checklist**

Developed from: Tong A, Sainsbury P, Craig J. Consolidated criteria for reporting qualitative research (COREQ): a 32-item checklist for interviews and focus groups. International Journal for Quality in Health Care. 2007. Volume 19, Number 6: pp. 349 – 357

| 0No. Item | Guide questions/description | Reported on Page # |
| --- | --- | --- |
| **Domain 1: Research team and reﬂexivity** |  |  |
| Personal Characteristics |  |  |
| 1. Interviewer/facilitator | Which author/s conducted the interview or focus group? | P3-4  Data Collection |
| 2. Credentials | What were the researcher’s credentials? E.g. PhD, MD | P4  Rigor and reflexivity |
| 3. Occupation | What was their occupation at the time of the study? | P4  Rigor and reflexivity |
| 4. Gender | Was the researcher male or female? | P4  Rigor and reflexivity |
| 5. Experience and training | What experience or training did the researcher have? | P4  Rigor and reflexivity |
| Relationship with participants |  |  |
| 6. Relationship established | Was a relationship established prior to study commencement? | P4  Rigor and reflexivity/ Ethical Consideration |
| 7. Participant knowledge of the interviewer | What did the participants know about the researcher? e.g. personal goals, reasons for doing the research | P4  Ethical Consideration |
| 8. Interviewer characteristics | What characteristics were reported about the inter viewer/facilitator? e.g. Bias, assumptions, reasons and interests in the research topic | P4  Rigor and reflexivity |
| **Domain 2: study design** |  |  |
| Theoretical framework |  |  |
| 9. Methodological orientation and Theory | What methodological orientation was stated to underpin the study? e.g. grounded theory, discourse analysis, ethnography, phenomenology, content analysis | P3  Study Design |
| Participant selection |  |  |
| 10. Sampling | How were participants selected? e.g. purposive, convenience, consecutive, snowball | P3  Setting and Participants |
| 11. Method of approach | How were participants approached? e.g. face-to-face, telephone, mail, email | P3  Setting and Participants/Data Collection |
| 12. Sample size | How many participants were in the study? | P3  Setting and Participants |
| 13. Non-participation | How many people refused to participate or dropped out? Reasons? | P3  Setting and Participants |
| Setting |  |  |
| 14. Setting of data collection | Where was the data collected? e.g. home, clinic, workplace | P3  Data Collection |
| 15. Presence of non-participants | Was anyone else present besides the participants and researchers? | P3  Data Collection |
| 16. Description of sample | What are the important characteristics of the sample? e.g. demographic data, date | P3/5  Setting and Participants /Results |
| Data collection |  |  |
| 17. Interview guide | Were questions, prompts, guides provided by the authors? Was it pilot tested? | P3  Data Collection |
| 18. Repeat interviews | Were repeat inter views carried out? If yes, how many? | P3  Data Collection |
| 19. Audio/visual recording | Did the research use audio or visual recording to collect the data? | P3  Data Collection |
| 20. Field notes | Were ﬁeld notes made during and/or after the interview or focus group? | P3  Data Collection |
| 21. Duration | What was the duration of the inter views or focus group? | P3  Data Collection |
| 22. Data saturation | Was data saturation discussed? | P3  Setting and Participants |
| 23. Transcripts returned | Were transcripts returned to participants for comment and/or correction? | / |
| **Domain 3: analysis and ﬁndings** |  |  |
| Data analysis |  |  |
| 24. Number of data coders | How many data coders coded the data? | P4  Data analysis |
| 25. Description of the coding tree | Did authors provide a description of the coding tree? | P4  Data analysis |
| 26. Derivation of themes | Were themes identiﬁed in advance or derived from the data? | P4  Data analysis |
| 27. Software | What software, if applicable, was used to manage the data? | P4  Data analysis |
| 28. Participant checking | Did participants provide feedback on the ﬁndings? | / |
| Reporting |  |  |
| 29. Quotations presented | Were participant quotations presented to illustrate the themes/ﬁndings? Was each quotation identiﬁed? e.g. participant number | P4-8  Results |
| 30. Data and ﬁndings consistent | Was there consistency between the data presented and the ﬁndings? | P4-8  Results |
| 31. Clarity of major themes | Were major themes clearly presented in the ﬁndings? | P4-8  Results |
| 32. Clarity of minor themes | Is there a description of diverse cases or discussion of minor themes? | P4-8  Results |

**Appendix 2:  Interview Guide**

1. How did you understand nutrition during your hospitalization in the transplant unit?
2. What were your experiences of eating and nutrition management during that period?
3. What symptoms or physical discomfort affected your eating the most, and how?
4. Did the transplant-unit environment or dietary restrictions affect your eating? If so, in what ways?
5. When eating became difficult, how did you cope or adjust your eating?
6. What roles did healthcare professionals and family members play in your nutrition management?
7. Did your usual eating habits, taste preferences, or food-related beliefs influence how you understood nutrition during hospitalization?
8. What kind of nutritional support did you need or expect during transplant-unit hospitalization?
9. Is there anything else about nutrition management during transplant-unit hospitalization that you would like to add?

**Note：**Probing questions were used as needed to elicit further detail and clarification.
